# The novel GLP-1/GIP dual agonist DA3-CH improves rat type 2 diabetes through activating AMPK/ACC signaling pathway

**DOI:** 10.18632/aging.205118

**Published:** 2023-10-17

**Authors:** Jing Xu, Peng Chen, Dongzhi Wu, Qiang Zhou, Sijie Chen, Xiang Ding, Hongping Xiong

**Affiliations:** 1 Department of Endocrinology, Fuzhou Second Hospital, Fuzhou 350007, Fujian Province, China; 2 The Third Clinical Medical College, Fujian Medical University, Fuzhou 362002, Fujian Province, China; 3 Department of Internal Neurology, Fuzhou Second Hospital, Fuzhou 350007, Fujian Province, China; 4 Department of Orthopedics Institute, Fuzhou Second Hospital, Fuzhou 350007, Fujian Province, China

**Keywords:** T2DM, DA3-CH, AMPK, ACC

## Abstract

Background: Type 2 diabetes mellitus (T2DM) accounts for more than 95% of all diabetes. DA3-CH is a novel dual receptor agonist of glucagon like peptide-1 (GLP-1) and glucose dependent insulin stimulating polypeptide (GIP). The regulatory role of DA3-CH in T2DM has not been reported.

Methods: T2DM rat model was established successfully with high sugar and fat feed and streptomycin (STZ) induction. The mRNA and protein expression were measured with RT-PCR and western blotting. The apoptosis level in the pancreatic tissue was evaluated with Tunel staining. Blood glucose, fat, and oxidative stress indicators were measured.

Results: DA3-CH greatly improved T2DM symptoms by reducing blood glucose, blood fat, pancreatic tissue injury, apoptosis, and oxidative stress condition. The inactivation of Adenylate activated protein kinase (AMPK)/acetyl CoA carboxylase (ACC) signaling pathway in T2DM rats was promoted by DA3-CH. The influence of DA3-CH was significantly reversed by Com-C, the inhibitor of AMPK/ACC signaling pathway.

Conclusions: DA3-CH might improve T2DM through targeting AMPK/ACC signaling pathway. This study might provide a novel therapeutic strategy for the prevention and treatment of T2DM through targeting DA3-CH and AMPK/ACC signaling pathway.

## INTRODUCTION

Diabetes is a global medical problem. With the economic development, changes in lifestyle and aging of the population, the prevalence of diabetes continues to rise, and it has become the third chronic lifelong disease that seriously endangers human health after cardiovascular diseases and tumors [1]. According to the estimation of the World Health Organization, the number of diabetes patients worldwide will reach about 366 million by 2030 [2]. Type 2 diabetes mellitus (T2DM) is a polygenic genetic disease caused by the joint action of genes and environment, which mainly occurs in adults, accounting for more than 95% of all diabetes [3]. In the later stages of disease development, lesions can easily affect organs such as the kidneys, heart, liver, and eyes, causing chronic damage, dysfunction, and even failure of various organs, seriously endangering human health [4]. The specific pathogenesis of T2DM is still not completely clear. Many research results show that there is a close relationship between its pathogenesis and oxidative stress.

DA3-CH is a novel dual receptor agonist of glucagon like peptide-1 (GLP-1) and glucose dependent insulin stimulating polypeptide (GIP) [5]. The antioxidant effect of DA3-CH has been confirmed under different *in vitro* experimental conditions [6]. In myocardial cells stimulated by palmitate, GLP-1 can reduce the oxidative stress of cytoplasm and mitochondria, increase the expression of mitochondrial ATP synthase, reduce the leakage of creatine to extracellular media, and inhibit the oxidative damage of total DNA and mitochondrial DNA [7]. GIP can promote the formation of insulin vesicles and increase insulin release. In addition, it can also promote insulin synthesis and secretion through the phosphatidylinositol-3 kinase and protein kinase B pathways. However, there are few reports on the impact of DA3-CH on T2DM.

Adenylate activated protein kinase (AMPK) and acetyl CoA carboxylase (ACC) have been proved to be closely related with the occurrence, development, deterioration of Type 2 diabetes [8]. AMPK, known as a cellular energy regulator, is a key factor in regulating energy metabolism and participates in the endocrine metabolism processes of numerous target organs [9]. It can regulate glucose metabolism, lipid metabolism, and protein metabolism to maintain cellular energy homeostasis [10]. ACC plays a crucial role in the biosynthesis and metabolism of fatty acids and is a key regulator of fatty acid synthesis and oxidation pathways [11]. Therefore, regulating the expression of ACC often helps to improve fat deposition, and ACC can serve as a potential target for obesity and T2DM treatment [12]. However, if DA3-CH could improve rat type 2 diabetes through regulating AMPK/ACC signaling pathway has not been reported.

In this study, T2DM rat model was established successfully with high sugar and fat feed (67% normal food+10% lard+20% sucrose+2.5% cholesterol+ 0 5% sodium cholate) and streptomycin (STZ, 25 mg/kg) induction. Blood glucose, fat, and oxidative stress indicators were evaluated. We firstly demonstrate that DA3-CH alleviates T2DM through targeting AMPK/ACC signaling pathway. The inhibition of apoptosis in the pancreatic tissues by DA3-CH is proved in this research. Our study might provide a novel therapeutic strategy for the prevention and treatment of T2DM through targeting DA3-CH and AMPK/ACC signaling pathway.

## MATERIALS AND METHODS

### Establishment of T2DM animal model

Specific pathogens free (SPF) grade male SD rats (200 ± 20) g purchased from Charles River were used in this research. The rats were raised on the condition of 23± 2° C, 35% ± 5% humidity, ventilation rate of 10-20 times/h. After adaptive feeding for 7 days, the rats were randomly divided into the following groups, control, T2DM, T2DM+DA3-CH, T2DM+Metformin (Met), and T2DM+DA3-CH+Com-C, with 10 rats in each group. The rats in the control group were fed with regular feed. The rest were given high sugar and fat feed (composition: 67% normal food+10% lard+20% sucrose+2.5% cholesterol+ 0 5% sodium cholate) for 4 weeks. Then, STZ (25 mg/kg) was injected intraperitoneally to create a model, and STZ (25 25 mg/kg) was injected again one week later. The control group received intraperitoneal injection of the same dose of normal saline. Three days after intraperitoneal injection, blood glucose was measured using a blood glucose meter. The blood glucose over 16.7 mmol/L was believed to successfully established T2DM model. The rats in the group T2DM+DA3-CH were treated with DA3-CH (10 nmol/kg) once a day through intraperitoneal injection as described previously [13]. The rats in the group T2DM+Met were treated with Met (30 mg/kg) once a day through gavage. The rats in the group T2DM+DA3-CH+Com-C were treated with DA3-CH (10 nmol/kg) and Com-C (0.2 mg/kg) once a day through intraperitoneal injection. The rats in the control group and T2DM were treated with same amount normal saline through gavage. 12 weeks after feeding with high sugar and fat feed, the rats were sacrificed for detection.

### Measurement of blood glucose, HOMA-IR, body weight, food and water consumption

The animal weight, food consumption, and water consumption, were measured at a fixed time per week. The blood was collected at week 12. Before collecting blood, all animals ceased their feed supply after 22:00 the previous day, without cutting off water, and blood were collected at 9:00 am the next day. Blood glucose was measured with a glucose meter (Roche, US) through tail vein. HOMA-IR = (Fasting Plasma Glucose (mmol/L) × Fasting Serum Insulin (μU/mL))/22.5.

### Hematoxylin-eosin (HE) staining

After sacrificing animals, the pancreas was isolated. The dehydrated sample was embedded with paraffin. After pre-cooling at 20° C for 30 minutes, the tissues were cut into 5 μm. After dewaxing, the tissues were immersed in hematoxylin solution (#G1140, Solarbio, China) for 5 minutes and washed with water until no staining solution flows out. The sections were incubated with PBS for 5 minutes for returning to blue. Then, the sections were stained with eosin (#MB9898-3, Meilunbio, China) for 15 seconds. After decolorization with 95% ethanol for 10 seconds, tissues were washed with water. Tissues were immersed in xylene for transparency for 10 minutes. Neutral gum (#MB9899, Meilunbio) sealing was performed.

### Tunel staining

Tunel staining kit (#C1098, Beyotime, China) was used in this study. The tissues were prepared as described in part 2.3. The tissues were incubated with protease K working solution (#ST537-2g, Beyotime) in a 37° C incubator for 30 minutes. After washing with PBS 3 times for 5 minutes each time, the tissues were incubated with 3% hydrogen peroxide (#16J29C, Boster, China) for 20 minutes. Incubation with biotin labeling solution at 37° C for 60 minutes, and Streptavidin HRP working solution at room temperature for 30 minutes were performed. After washing with PBS 3 times for 5 minutes each time, tissues were incubated with DAB working at room temperature for 30 seconds, and stained with hematoxylin for 5 minutes. Dehydration with anhydrous ethanol for 7 minutes, and transparency with xylene were performed. After slicing and drying, neutral gum (#MB9899, Meilunbio) sealing was performed.

### Western blotting

The proteins were firstly lysed using RIPA lysis buffer (#MA0151, Meilunbio) containing protein phosphatase inhibitor (#MB12707, Meilunbio). The protein concentrations were measured using BCA assay (#23209, Thermo Fisher, USA). Same amount of protein (30 μg) was loaded for 10% SDS-PAGE, and transferred to a PVDF membrane. TBST containing 5% non-fat milk (#MB4219-3, Meilunbio) was used to block membrane for 2 h. Then, the proteins were incubated with primary antibodies at 4° C overnight. After washing with PBS, the membrane was incubated with secondary antibodies for 2 h. Then, membrane was measured using a hypersensitivity chemiluminescence detection kit (Baisai Biology, #S6009M) and observed with chemiluminescence imaging system (ChemiDoc Touch, Bio-Rad, USA), and ImageJ software was used to analyze protein band. The following antibodies were used in this research: rabbit monoclonal to p-AMPK (ab133448, Abcam, UK), rabbit monoclonal to p-acetyl CoA carboxylase (ab68191), rabbit polyclonal to GAPDH (ab9485), goat Anti-Rabbit (ab150077).

### Immunohistochemical staining

The tissues were heated in a microwave oven for 3 minutes for antigen repair, and incubated with 3% hydrogen peroxide for 2 minutes. After washing with PBS for 3 times (5 min/time), the sections were blocked with 5% non-fat milk (#MB4219-3, Meilunbio), and incubated with primary antibody. After 3 times washing with PBS (5 min/time), the second antibody was added to incubate sections for 3 h. Then, DAB chromogenic solution was added. After dehydration and mount, the sections were captured with Olympus BX41 microscope (Japan). The following antibodies were used in this research: Rabbit monoclonal to Bax (ab32503), goat Anti-Rabbit (ab150077). The calculation of IHC staining was performed using Image J software.

### Biochemical indicators detection

After sacrificing rats, blood was collected through abdominal aorta. The blood was centrifuged at 2000 g for 10 minutes, and serum was collected for detection of total cholesterol (#A111-1-1, Nanjing Jiancheng Bioengineering Institute, China), triglyceride (#A110-1-1, Nanjing Jiancheng Bioengineering Institute, China), high density lipoprotein (#A112-1-1, Nanjing Jiancheng Bioengineering Institute, China) with related commercial kits according to the instruction.

### Detection of redox system indicators

The serum was prepared as described in part 2.6. Glutathione peroxidase (GSH-PX, S0053, Beyotime, China), superoxide (SOD, S0101S, Beyotime, China), and MDA (malondiadehyde, S0131M, Beyotime, China) were detected with related commercial kits according to the instruction.

### RT-PCR

Trizol (#R0016, Beyotime) was used to extract RNA from tissues. RNA purity was measured with Nanodrop 2000 spectrophotometer (Thermo Scientific, USA). Takara PrimeScript RT reagent kit with gDNA eraser kit (#RR047A) was used for reverse transcription. RT-PCR was performed with Bio-Rad (CXF96). The relative expression level of gene was measured through 2^-ΔΔCT^ method. The primers are listed as follows: AMPK (F: CAGAGGACACTATGTCTGG, R: GCTTGGGATTGAGGACT), ACC (F: GTCTGCTGGGAAGTTAATCCAG, R: TCCTGCAGCTCTAGCAGAGG), β-actin (F: TGGCACCCAGCACAATGAA, R: CTAAGTCATAGTCCGCCTAGAAGCA).

### Statistical analysis

SPSS20.0 software was used for statistical analysis. The data were presented with mean ± standard deviation. ANOVA was used to analyze values among multiple groups, and t-test was used to analyze the results of two groups. p < 0.05 indicate statistical differences.

### Availability of data and material

The data and material used to support the findings of this study are included within the manuscript and Supplementary Files.

## RESULTS

### DA3-CH greatly improves T2DM symptoms

**Figure 1:**
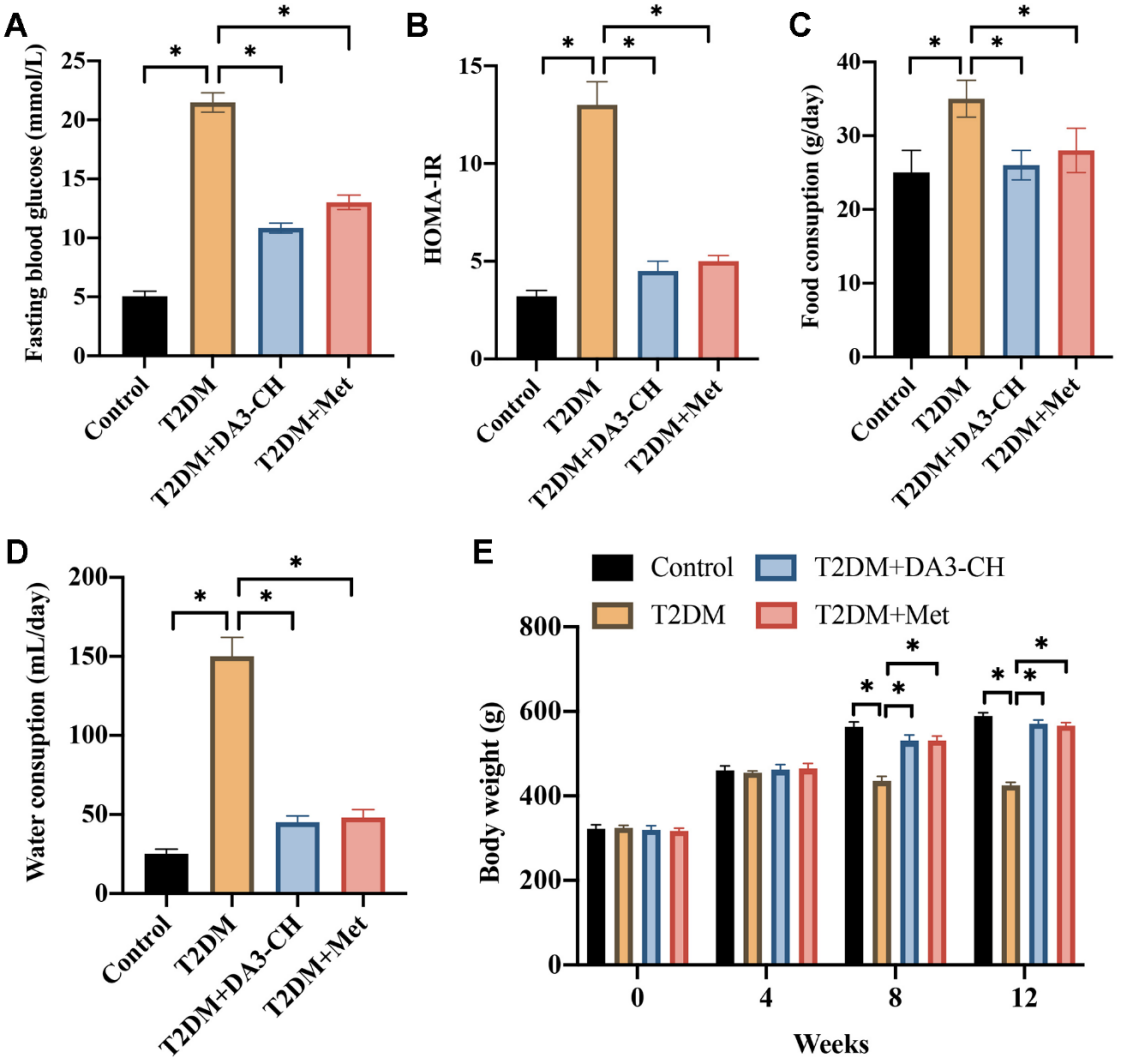
**DA3-CH greatly improves T2DM symptoms.** (**A**) The level of fasting blood glucose was measured; (**B**) The HOMA-IR was analyzed; (**C**) The food consumption was calculated; (**D**) The water consumption was calculated; (**E**) The body weight was recorded. * means p <0.05. n=5 for each group.

To investigate the regulatory role of DA3-CH in T2DM. The T2DM rat model was established successfully. Significant higher fasting blood glucose (Figure 1A), HOMA-IR (Figure 1B), food (Figure 1C) and water consumption (Figure 1D), but lower weight (Figure 1E) was observed in the group T2DM. However, both treatment with DA3-CH and Met could lessen T2DM symptoms by reducing fasting blood glucose (Figure 1A), HOMA-IR (Figure 1B), food (Figure 1C) and water consumption (Figure 1D), but increasing weight (Figure 1E). These findings suggest that DA3-CH presents protective effects on T2DM.

### Significant tissue injury and apoptosis in pancreatic tissues of T2DM rats were inhibited by DA3-CH

**Figure 2:**
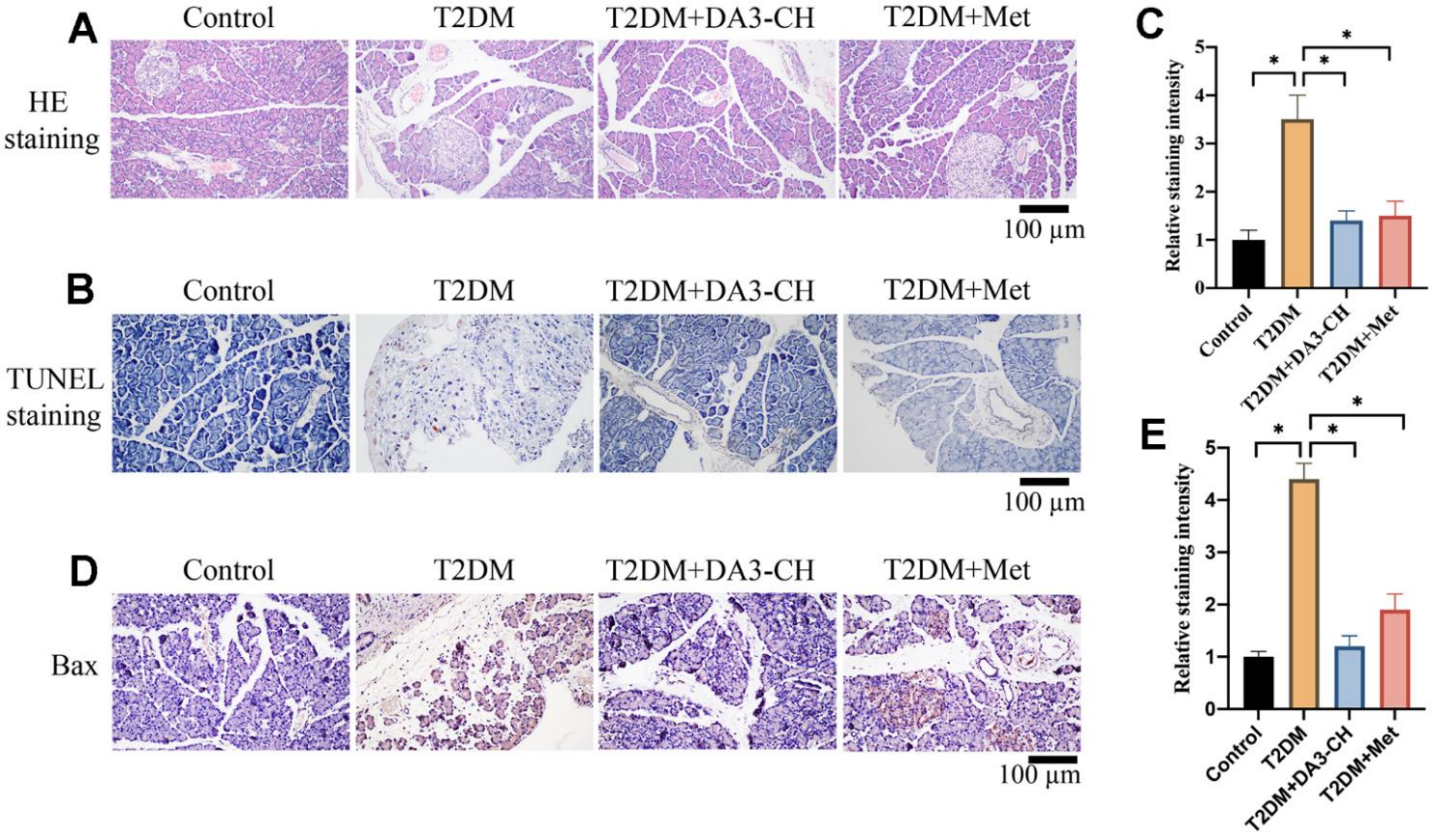
**Significant tissue injury and apoptosis in pancreatic tissues of T2DM rats were inhibited by DA3-CH.** (**A**) HE staining was performed to investigate the influence of DA3-CH on pancreatic tissue injury *in vivo*; (**B**) Tunel staining was performed to investigate the influence of DA3-CH on apoptosis level *in vivo*; (**C**) The staining intensity was analyzed; (**D**) IHC staining was performed to investigate the influence of DA3-CH on Bax expression *in vivo*; (**E**) The staining intensity was analyzed; * means p <0.05. n=5 for each group.

HE, Tunel, and IHC staining were performed to investigate the influence of DA3-CH on pancreatic tissue injury, apoptosis level, and Bax expression *in vivo*. Increased tissue gap and disordered arrangement in the group T2DM were improved by DA3-CH and Met (Figure 2A). In addition, remarkable increase of apoptosis in pancreatic tissue (Figure 2B, 2C) and higher expression of Bax (Figure 2D, 2E) in T2DM rats were suppressed by DA3-CH, indicating that DA3-CH could suppress the pancreatic tissue injury and apoptosis *in vivo*.

### DA3-CH markedly inhibited the blood fat levels and oxidative stress condition of T2DM rats

**Figure 3:**
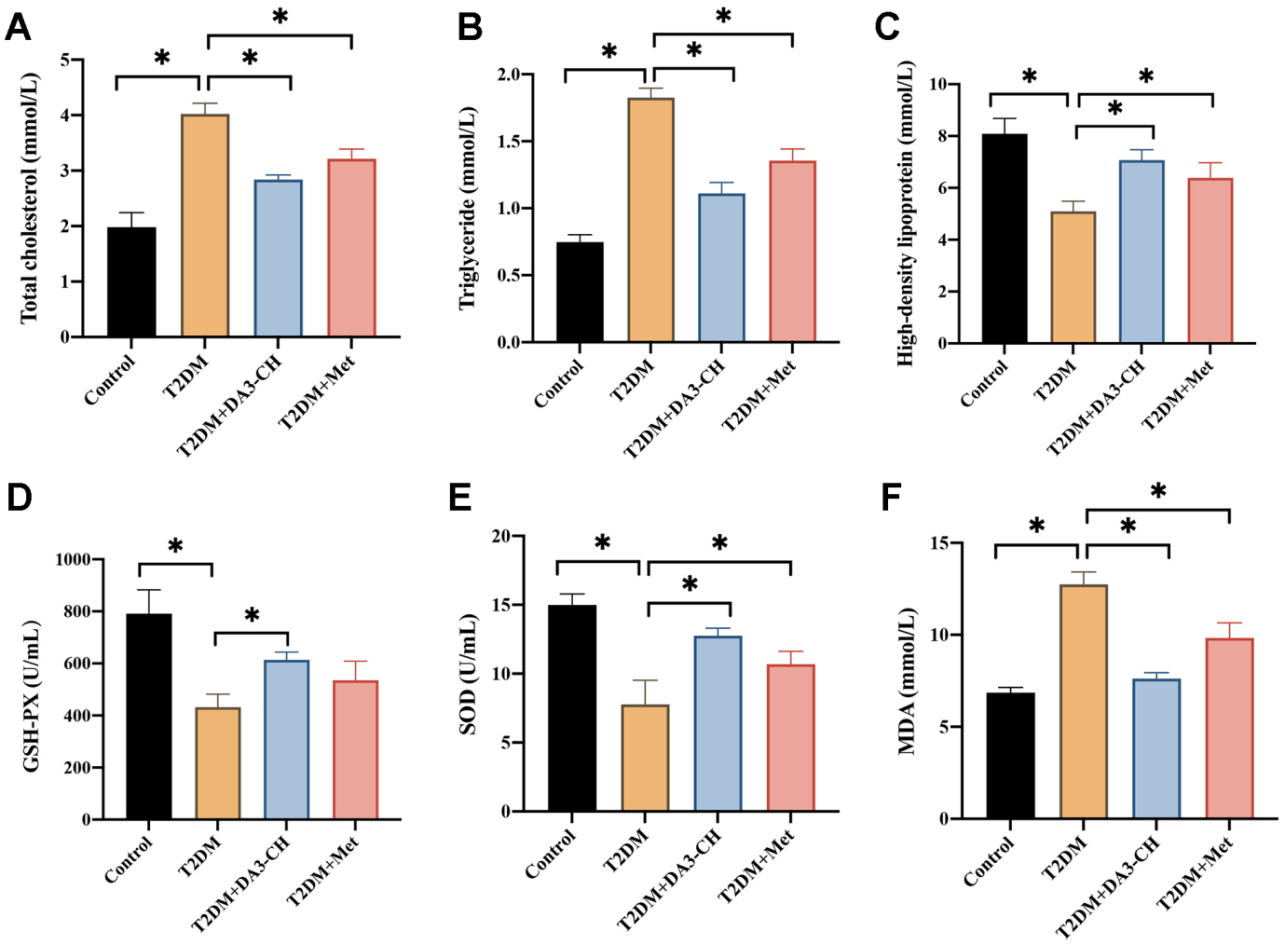
**DA3-CH markedly inhibited the blood fat levels and oxidative stress condition of T2DM rats.** (**A**) The total cholesterol level in the serum was measured; (**B**) The triglyceride level in the serum was measured; (**C**) The high-density lipoprotein in the serum was measured; (**D**) The GSH-PX level in the serum was measured; (**E**) The SOD level in the serum was measured; (**F**) The MDA level in the serum was measured. * means p <0.05. n=3 for each group.

Meanwhile, we found that DA3-CH could improve the blood fat level by reducing total cholesterol (Figure 3A), triglyceride (Figure 3B), but increasing high density lipoprotein (Figure 3C). In addition, DA3-CH greatly inhibited the oxidative stress condition by promoting GSH-PX (Figure 3D), SOD (Figure 3E), but reducing MDA (Figure 3F) levels in the serum.

### The inactivation of AMPK/ACC signaling pathway in T2DM rats was promoted by DA3-CH

**Figure 4:**
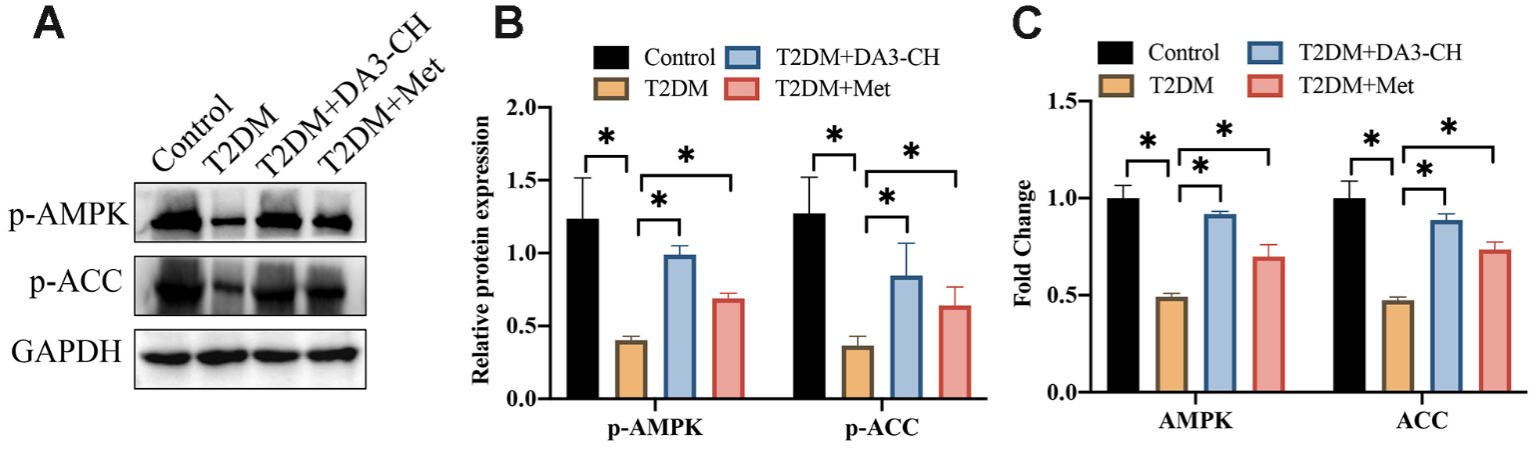
**The inactivation of AMPK/ACC signaling pathway in T2DM rats was promoted by DA3-CH.** (**A**) The protein levels of p-AMPK and p-ACC were measured with western blotting; (**B**) The protein expression was analyzed; (**C**) The mRNA levels of AMPK and ACC were detected with RT-PCR. * means p <0.05. n=3 for each group.

AMPK/ACC signaling pathway has been believed to closely linked with the progression of T2DM. We found that AMPK/ACC signaling pathway was suppressed in T2DM rats compared with control group (Figure 4A–4C). However, DA3-CH or Met greatly activated AMPK/ACC signaling pathway.

### Inactivation of AMPK/ACC signaling pathway by Com-C significantly reversed the influence of DA3-CH on T2DM

**Figure 5:**
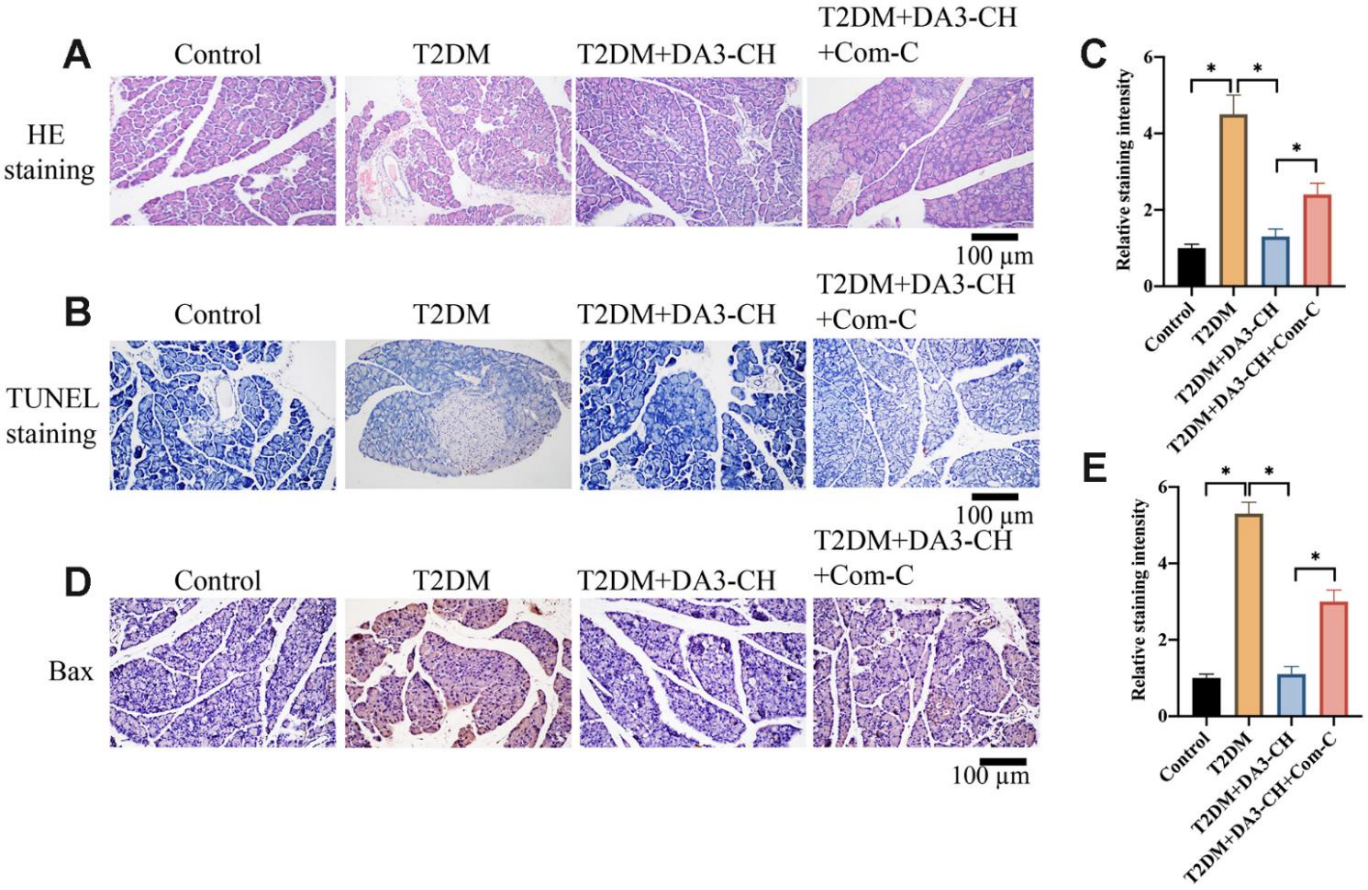
**Inactivation of AMPK/ACC signaling pathway by Com-C significantly reversed the influence of DA3-CH on pancreatic injury.** (**A**) HE staining was performed to investigate the influence of DA3-CH and Com-C on pancreatic tissue injury *in vivo*; (**B**) Tunel staining was performed to investigate the influence of DA3-CH and Com-C on apoptosis level *in vivo*; (**C**) The staining intensity was analyzed; (**D**) IHC staining was performed to investigate the influence of DA3-CH and Com-C on Bax expression *in vivo*; (**E**) The staining intensity was analyzed; * means p <0.05. n=3 for each group.

**Figure 6:**
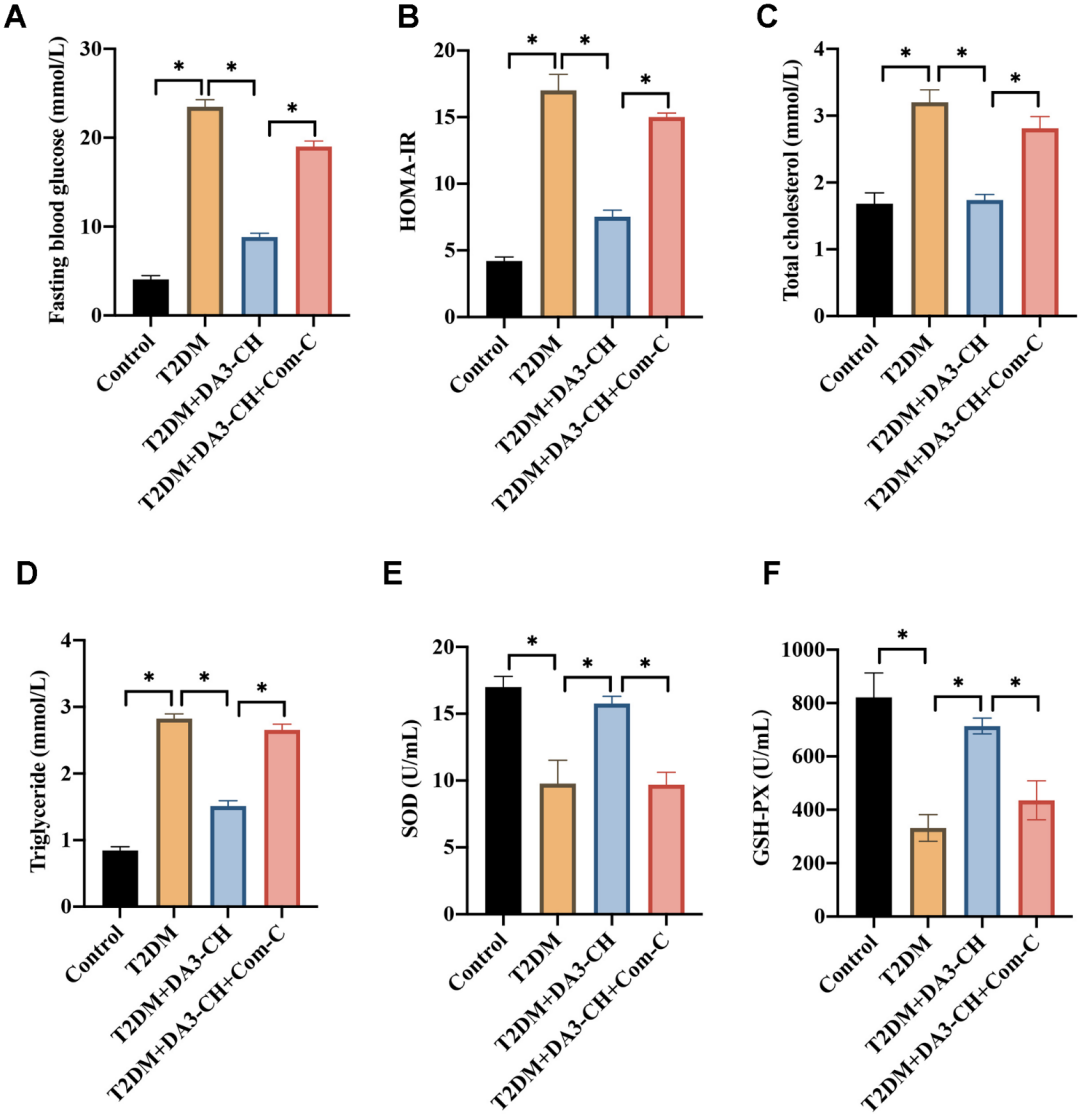
**Inactivation of AMPK/ACC signaling pathway by Com-C significantly reversed the influence of DA3-CH on fat levels and oxidative stress condition.** (**A**) The level of fasting blood glucose was measured; (**B**) The HOMA-IR was analyzed; (**C**) The total cholesterol level in the serum was measured; (**D**) The triglyceride level in the serum was measured; (**E**) The SOD level in the serum was measured; (**F**) The GSH-PX level in the serum was measured. * means p <0.05. n=5 for each group.

To investigate if DA3-CH improved T2DM through targeting AMPK/ACC signaling pathway, compound C (Com-C), the inhibitor of AMPK/ACC signaling pathway, was used to treat T2DM. We found that Com-C could reverse the influence of DA3-CH in T2DM rats by aggravating pancreatic tissue damage (Figure 5A), promoting tissue apoptosis (Figure 5B, 5C), and increasing Bax expression (Figure 5D, 5E). Meanwhile, Com-C reversed the influence of DA3-CH on glucose, blood fat and oxidative stress indicator level by increasing fast blood glucose (Figure 6A), HOMA-IR (Figure 6B), total cholesterol (Figure 6C), triglyceride (Figure 6D), but decreasing SOD (Figure 6E) and GSH-PX (Figure 6F). These findings suggest that DA3-CH might improve T2DM through activating AMPK/ACC signaling pathway.

## DISCUSSION

GLP-1 is secreted by L cells in the lower segment of the small intestine, also known as incretin [14]. Its main biological function is to stimulate glucose mediated insulin synthesis, secretion, and inhibit glucagon secretion in β cells. GLP-1 can increase the proliferation and insulin secretion of β cells [15]. DA3-CH is the agonist of GLP-1.

Studies have shown that DA3-CH has a significant protective effect on cerebral ischemia-reperfusion injury with diabetes, which can improve neurological defects, reduce the area of cerebral infarction, and reduce the expression of inflammatory factors in the acute phase of cerebral ischemia-reperfusion injury [13, 16]. However, the effect of DA3-CH on the function of pancreatic tissue in T2DM rats has not been reported. Meanwhile, they didn’t systematically investigate the inhibition effect of DA3-CH on T2DM. In this research, we firstly demonstrated that DA3-CH alleviated the pancreatic tissue injury and apoptosis of T2DM rats.

The activation of AMPK can affect the biological functions of cells, such as cell proliferation, apoptosis, growth, autophagy, and mitochondrial regulation [17]. At the same time, AMPK is also involved in obesity Metabolic syndrome and other metabolic diseases play an important role in the prevention and treatment of tumors, and can serve as potential drug targets, providing new ideas and prospects for drug research and development [18, 19].

ACC plays an important role in metabolic diseases. After treatment with ACC inhibitor, increased fatty acid oxidation, decreased fat triglycerides, increased insulin sensitivity, and reversed liver steatosis were observed in high-fat fed rats [20, 21]. However, the role of AMPK/ACC in T2DM is seldom reported. We found that AMPK/ACC signaling pathway was inhibited in T2DM, but was activated by DA3-CH and Met treatment.

## CONCLUSIONS

In summary, we proved that DA3-CH could greatly improve T2DM symptoms by reducing blood glucose, blood fat, pancreatic tissue injury, apoptosis, and oxidative stress condition. However, the influence of DA3-CH was significantly reversed by Com-C, the inhibitor of AMPK/ACC signaling pathway. DA3-CH might improve T2DM through targeting AMPK/ACC signaling pathway.
